# Surgical Management of Chronic Rhinosinusitis in Cystic Fibrosis

**DOI:** 10.3390/medsci7040057

**Published:** 2019-04-07

**Authors:** Zhong Zheng, Chetan Safi, David A. Gudis

**Affiliations:** Department of Otolaryngology—Head and Neck Surgery, Columbia University Irving Medical Center, New York, NY 10032, USA; zz2618@cumc.columbia.edu (Z.Z.); cys9028@nyp.org (C.S.)

**Keywords:** rhinosinusitis, cystic fibrosis, endoscopic sinus surgery, medial maxillectomy

## Abstract

Cystic fibrosis patients frequently develop chronic rhinosinusitis as a result of their propensity to form inspissated mucus and impairment of mucociliary clearance. They exhibit variable symptom burden even in the setting of positive radiographic and endoscopic findings. Current evidence suggests a positive effect of managing sinonasal disease on pulmonary health. Topical antimicrobial and mucolytic therapies are frequently required to manage the disease with surgery reserved for refractory cases. Endoscopic sinus surgery has been demonstrated to be safe and efficacious in controlling symptoms of chronic rhinosinusitis in patients with comorbid cystic fibrosis. However, the impact of surgery on pulmonary health remains an active area of investigation. In addition, a growing body of research has suggested a more extended surgical approach creating large sinonasal cavities with gravity-dependent drainage pathways, followed by adjuvant medical therapies, as an ideal strategy to optimally control disease and prevent pulmonary exacerbations. In this manuscript, we provide an up-to-date review of current evidence in the surgical management of chronic rhinosinusitis in cystic fibrosis patients.

## 1. Introduction

Cystic fibrosis (CF) is an autosomal recessive disease characterized by a defect in the cystic fibrosis transmembrane conductance regulator (CFTR) gene on chromosome 7, which encodes for a chloride ion transporter on the apical membrane of epithelial cells. It predominantly affects the Caucasian population, with a prevalence of ~30,000 patients in the United States and ~70,000 patients worldwide [1]. As a result of defective trans-membrane anion transport, CF patients develop increased epithelial sodium and water resorption and produce thick inspissated secretions, which then cause mucociliary stasis, chronic inflammation, bacterial colonization, and infection in multiple organ systems, including the upper and lower airways. The average life expectancy of CF patients is 48.5 years, and the majority of CF-related mortalities are due to progressive pulmonary decline, with many patients eventually requiring lung transplantation [2,3].

Impaired sinonasal mucociliary clearance mechanisms predispose CF patients to develop chronic rhinosinusitis (CRS) and nasal polyposis. On computed tomography (CT), CF patients also have under-developed paranasal sinuses due to chronic inflammation, further complicating ventilation and drainage [4]. Furthermore, a growing body of research has demonstrated a significant link between sinonasal and pulmonary health with regards to bacterial colonization, with an 80% concordance of bacterial isolates from sinonasal and bronchoalveolar lavages (BAL) [5]. The mainstays of medical therapies for CF CRS include oral and topical antibiotics directed at *Pseudomonas aeruginosa* and *Staphyloccocus aureus* species, topical mucolytic agents, and more recently CFTR modulators [6]. Refractory to medical therapy, endoscopic sinus surgery (ESS) has been shown to improve sinonasal symptoms and quality of life outcomes and reduce pulmonary bacterial colonization [5,7]. However, currently available data on the effect of ESS on pulmonary functions are conflicting [5,8]. In this manuscript, our objective is to provide an up-to-date review of current evidence in the surgical management of CRS in CF patients.

## 2. Unified Airway Health

Drawing a parallel with the unified airway model in reactive airway disease, mounting evidence has demonstrated a significant correlation between the health of paranasal sinuses and lungs. Studies have shown a possible mechanism of bacterial translocation from upper to lower airways. Therefore, in addition to alleviating symptoms of CRS in CF, ESS has the added benefit of creating an open and accessible sinonasal cavity to topical therapies and reducing bacterial seeding of the lungs. Furthermore, the reduction or eradication of pulmonary pathogenic colonization is critically important to prevent allograft rejection in post-lung transplant CF patients. A recent study compared microbiota composition from the sinuses and lung brushings using 16s RNA sequencing techniques, and microbiome diversity was found to be diminished in both the sinuses and lungs in CF CRS patients. Interestingly, non-CF CRS patients had distinct niches of microorganisms in their upper versus lower airways, while CF CRS patients had indistinguishable niches at both anatomic sites, lending further evidence to the theory of sinonasal cavity as a reservoir for bacterial translocation to lower airway in CF patients [9]. Walter and colleagues followed a cohort of 11 CF patients pre- and post-lung transplant, and identical *P. aeruginosa* isolates were seen in all patients within the sinuses and lungs [10]. Moreover, Mainz et al evaluated 182 CF patients and demonstrated significant genotypic concordance of *P. aeruginosa* and *S. aureus* isolates in upper and lower airways. Genetically identical strains of *P. aeruginosa* and *S. aureus* were identified in 31 of 36 and 23 of 24 patients, respectively. In addition, patients with positive *P. aeruginosa* sputum cultures were 88 times more likely to be colonized in the upper airway with the same bacterial pathogen [11]. In a different study, the same authors also found rapid colonization of new donor lungs with *P. aeruginosa* that was genetically identical to pre-transplant isolates within four weeks post-transplantation, and this colonization could be prevented by topical colistin antibiotic therapy [12]. A different group demonstrated the median time of recovery of *P. aeruginosa* species from CF lung transplant patients was 15 days post -peratively, as compared to 158 days in non-CF recipients. Histologically, evidence of *Pseudomonas* infection was also detected earlier at post-operative day (POD) #10, (vs. POD #261 in non-CF) and at a higher rate (13/44 in CF vs. 3/21 in non-CF) [13]. Overall, although currently available evidence is limited by its retrospective nature and mostly small case series, with many including both surgical and medical controls; however, a correlation between sinonasal and pulmonary health with regards to pathogenic bacterial colonization has been suggested. Thus, ESS plays an important role in reducing the bacterial burden in the upper airway to prevent subsequent seeding of the lower airway. 

## 3. Surgical Management

Although almost all CF patients have endoscopic and radiological findings of sinonasal disease and a majority of them have extensive nasal polyposis, patient-reported subjective symptoms are variable at less than 20% [2]. Despite appropriate medical therapy, 20 to 60% of CF patients go on to require ESS [2]. In a retrospective review, Brook et al. showed that prior history of ESS and severe CFTR mutations are predictive of ESS while Sinonasal Outcome Test (SNOT)-22 score was not [14]. In a different study, CF patients with nasal polyposis, prior history of ESS, lower forced expiratory volume in one second (FEV1), higher Lund-Mackay score, and higher SNOT-22 score was more likely to elect up-front ESS versus medical therapy. Furthermore, a delay in surgery did not affect post-operative improvement [15]. There are no current guidelines specifically for the management of CF CRS, and patient selection for ESS should follow previously established recommendations for the management of CRS [16] with the additional consideration for reducing pulmonary pathogen colonization, especially in post-lung transplant CF patients.

Radiographically, CF patients have a high prevalence of frontal aplasia, and maxillary, ethmoid, and sphenoid hypoplasia. Sclerotic sphenoethmoidal partitions are also commonly found. Interestingly, other important anatomic variants were seen differentially in CF patients: Haller cells and concha bullosa were rarely seen, whereas Onodi cells were more frequently observed [4,17]. Careful pre-operative evaluation of CT findings is critical in avoiding complications and ensuring complete removal of all bony partitions. Complete ESS is especially important in CF CRS, as inspissated secretions may be trapped in partially removed partitions. A recent study also demonstrated high-risk CF mutations are associated with more severe radiographic findings as measured by modified Lund–Mackay scores, as well as higher prevalence of sinus hypoplasia/aplasia [18]. An understanding of CF pathophysiology is important in the surgical management of CF CRS patients to ensure surgical success. In our experience, intra-operative image guidance can be an invaluable tool due to anatomic differences in CF patients, especially in revision cases. However, its use should be judicious and always correlated with knowledge of surgical landmarks. Lastly, a cohort of pediatric CF patients who underwent ESS during and after facial growth spurts was followed prospectively for over 10 years, and no significant differences in cephalometric measurements were demonstrated, which corroborates previous findings on the safety of ESS on pediatric facial growth [19].

Large systematic reviews have demonstrated a definitive benefit of ESS on patient-reported quality of life (QoL) outcomes, while the effect of sinus surgery on pulmonary function tests (PFTs) have yielded conflicting results to date. Khalid et al. demonstrated significant improvement in QoL as measured by the Rhinosinusitis Disability Index (RSDI) in CF patients after ESS that was comparable to control patients, even though CF patients had worse pre-operative CT and endoscopic findings [20]. In one systematic review, Macdonald et al. found that ESS consistently improved sinonasal symptoms in CF patients, and some evidence existed for reduced days of hospitalization for pulmonary exacerbations and usage of intravenous antibiotic therapy. However, no significant changes in pulmonary function tests, including FEV1, were identified [21,22]. Another systematic review similarly demonstrated that ESS led to improved sinonasal symptoms and endoscopic scores, while PFTs were improved in 3/8 level four studies [5]. Kovell et al. demonstrated an improvement in PFTs in pediatric CF patients following ESS, although some of the benefits were mitigated by lower socioeconomic status [23]. Khalfoun and colleagues found that the decline in FEV1 was prevented by ESS in patients with moderate to severe lung disease [8]. Large systematic reviews evaluated the entire aggregate of CF patients; subgroup analysis of high versus low-risk mutations may provide additional insights and can be an area of future research. A study by Halderman and colleagues alluded to this and demonstrated differential responses of PFTs to ESS in homozygous versus heterozygous F5018del CF patients [24]. 

Endoscopic sinus surgery also plays a critical role in reducing or eradicating pulmonary colonization of pathogens in CF patients. Aanaes and colleagues prospectively followed a cohort of 106 CF patients after ESS with adjuvant systemic and topical therapy, and they demonstrated reduced pulmonary colonization with CF pathogens at 6 months [7]. Furthermore, the percentage of patients without any gram-negative bacteria colonization in the lungs increased from 15% to 33% at 3 years. Although overall pulmonary decline was observed for the cohort, a small percentage of patients had stable lung functions [25]. Holzmann et al. studied the effects of ESS on 37 post-lung transplant CF patients and found a significant correlation between sinus culture positivity and BAL positivity. Successful ESS (defined as less than 3 post-operative sinus aspirates with significant bacterial growth) was associated with significantly reduced incidence of lower airway exacerbations and a trend toward decreased bronchiolitis obliterans syndrome (BOS) [26]. Similarly, Vital et al. demonstrated a significant correlation between chronic sinonasal colonization and lung allograft infection rates in CF patients after lung transplantation and sinus surgery. Furthermore, ESS and daily saline irrigation were successful in eliminating *P. aeruginosa* colonization in more than a third of patients [27]. The absence of persistent airway pathogenic colonization was associated with less frequent and delayed development of BOS and increased overall survival [28]. In contrast, Leung and colleagues found that pre-transplant ESS did not prevent post-transplant *Pseudomonas* re-colonization and did not affect overall survival [29]. 

## 4. Extended Sinus Surgery

Recently, modified endoscopic medial maxillectomy (MEMM) has been increasingly performed for recalcitrant maxillary sinus disease. This technique is especially useful in CF patients, allowing large cavities for gravity-dependent drainage, improved topical medication delivery, and access for office-based debridement or polypectomy. A recent study combining MEMM with mucosal stripping of maxillary sinuses and total ethmoidectomy showed significant volume reduction of maxillary sinuses through osteoneogenesis, which the authors proposed as a mechanism for decreased mucus accumulation, thus, chronic bacterial colonization of the sinonasal cavity [30]. The sustained improvement of sinonasal symptoms after MEMM has been demonstrated for up to 6.9 years, although the patient population in this study was more heterogeneous and included all patients with recalcitrant chronic maxillary sinusitis [31].

According to Shatz, a combined Caldwell–Luc approach and medial maxillectomy for CF CRS patients with prior ESS failure were efficacious in reducing episodes of hospitalizations and need for IV antibiotics. Interestingly, FEV1 was significantly improved at 6 months in his cohort of 15 patients [32]. Virgin and colleagues followed a cohort of 22 patients after MEMM and found significant improvement in patient-reported symptom scores, as well as a decreased number of pulmonary exacerbations requiring hospitalization with 1 year follow up [1]. 

CF patients often have aplastic or hypoplastic frontal sinuses; however, the modified endoscopic Lothrop procedure is a consideration in recalcitrant frontal sinus disease in CF patients. Jaberoo et al. described a series of two CF patients who safely underwent modified Lothrop procedure with good symptomatic improvement [33]. Although the safety and efficacy can be extrapolated from non-CF CRS literature, larger prospective studies are needed to evaluate outcomes of Draf 3 procedures in CF CRS patients. 

## 5. Post-Operative Adjuvant Therapy

Similar to our understanding of non-CF CRS, a combined approach of endoscopic sinus surgery followed by post-surgical adjuvant medical therapy represents the most optimal treatment for disease control in CF CRS. Aanaes et al. demonstrated that intensive post-operative nasal saline irrigation, topical colistin, and office debridement following ESS eradicated pathogenic bacteria in 67% of operated sinuses 6-months post-operatively, with effect sustained up to 3 years [34]. Similarly, the frequency of BAL negativity for CF pathogens increased by 150% 1 year after sinus surgery with adjuvant therapy [7]. Moss and colleagues studied the effect of topical tobramycin irrigation after ESS and found a reduction in the need for future surgical interventions [35]. Combining complete ESS with MEMM and a post-operative regimen that included both oral and topical antibiotic and steroid therapy, Virgin et al. showed a significant reduction of symptoms and hospitalizations for pulmonary exacerbations [1]. Cimmino and colleagues performed a small randomized double-blind placebo-controlled trial of 24 CF patients who underwent sinus surgery and then maintained on dornase alfa therapy, and they demonstrated a significantly improved FEV1 compared to control patients. In addition, patient-reported symptom scores and Lund–Kennedy endoscopic scores were both improved over placebo [36].

## 6. Summary

The treatment of chronic rhinosinusitis with comorbid cystic fibrosis is complex and challenging. High disease burden found on endoscopic or radiographic examination often does not correlate with patient self-reported symptoms. Currently available data are limited to mostly case series, and further larger prospective studies are much-needed. Endoscopic sinus surgery has been shown to improve sinonasal and pulmonary bacterial colonization, as well as alleviating patient symptoms. The effects of sinus surgery on pulmonary functions are less clear. Despite a paucity of high-quality data and lack of an established treatment algorithm, increasing research has suggested that a multi-disciplinary approach with extensive sinus surgery creating gravity dependent drainage pathways, combined with adjunct topical and medical therapies, offer the most optimal treatment strategy for CF CRS patients.
